# Editorial: Neural Mechanisms of Memory Retrieval and Its Links to Other Cognitive Processes

**DOI:** 10.3389/fnhum.2021.810073

**Published:** 2021-12-16

**Authors:** Sue-Hyun Lee, Xiaonan L. Liu, Marc N. Coutanche

**Affiliations:** ^1^Department of Bio and Brain Engineering, College of Engineering, Korea Advanced Institute of Science and Technology (KAIST), Daejeon, South Korea; ^2^Program of Brain and Cognitive Engineering, College of Engineering, Korea Advanced Institute of Science and Technology (KAIST), Daejeon, South Korea; ^3^Center for Neuroscience, University of California, Davis, Davis, CA, United States; ^4^Department of Psychology, University of California, Davis, Davis, CA, United States; ^5^Department of Psychology, University of Pittsburgh, Pittsburgh, PA, United States; ^6^Learning Research and Development Center, University of Pittsburgh, Pittsburgh, PA, United States

**Keywords:** memory, retrieval, cognitive load, testing effect, reconsolidation, AMPA receptor

Memory retrieval is an active process. Acquired memories can be modified, altered, or strengthened through online retrieval. Moreover, memory retrieval is closely related to other cognitive processes, such as working memory and memory consolidation. The papers included in this Research Topic examine the role of memory retrieval in memory strengthening and reconsolidation, factors affecting memory retrieval, and the cellular mechanisms underlying retrieval. In this Editorial, we provide an overview of the contents of this Research Topic.

## Testing Effects

As many students can attest, a practice test is an excellent way to form long-term memories. Why is a retrieval test so effective compared to directly studying the same material? Marin-Garcia et al. ask this by measuring brain activity with fMRI as people retrieved Swahili-English word pairs that they had learned one week before. By randomly assigning participants to a study or test group, the researchers were able to ask how the retrieving brain differs, based on this learning history. The test group showed the expected boost to retrieval performance, and both groups activated memory-related regions within and outside the medial temporal lobe (Buckner et al., 1998). But how did their neural activity differ? A history of testing, compared to studying, led to greater activation in the left putamen and left inferior parietal cortex for remembered, rather than forgotten, word pairs. The putamen's involvement is consistent with the use of procedural speech planning and production processes, suggesting that greater interactions between memory systems may underlie post-test retrieval. The study group instead demonstrated stronger activation across frontal regions for remembered word pairs, which, intriguingly, was the pattern shown for forgotten word pairs in the test group. Marin-Garcia et al. hypothesized that this reflects greater top-down control: for both successfully remembered words after studying, and for attempts to retrieve difficult-to-remember word pairs after testing. Thus, different brain networks reflect how retrieved information is originally encoded, giving us the striking behavioral benefits of retrieval testing.

## Cognitive Loads

Sisakhti et al. examined the effects of cognitive load on memory retrieval of well-encoded images and underlying neural processes. Consistent with their prediction, self-rated memory retrieval performance declined as cognitive load increased. Using functional magnetic resonance imaging (fMRI), the authors also showed that retrieval of high-load images elicited stronger activation in certain brain regions, such as the parahippocampus, cerebellum, superior lateral occipital, fusiform and lingual gyri, precuneus, and posterior cingulate gyrus. These results support the notion that memory retrieval is actively and dynamically associated with other cognitive processes, rather than simply reproducing encoded information.

## AMPA Receptors

Moving from the system level to the synaptic level, Pereyra and Medina review the role of AMPA receptors in memory retrieval. Historically, this field has tended to focus on the role of AMPA receptors in encoding, meaning there is still much to learn about their role in retrieval. Rodent studies have provided important results in this area, however, which Pereyra and Medina review and discuss. The number, composition, and mobility of AMPA receptors are all important in memory retrieval, as is the timing of synaptic changes. Expanding our knowledge of these, and other AMPA properties is of interest to more than just basic science. The nature of AMPA receptors has important therapeutic consequences. As we remember, the retrieved memories are updated, giving potential for future inventions, particularly in people experiencing involuntary retrieval of past traumatic events in conditions such as posttraumatic stress disorder (PTSD). Realizing such benefits starts with developing the sort of understanding that Pereyra and Madina discuss.

## Reconsolidation

Memory reconsolidation theory suggests that retrieval itself can be a signal for a dynamic memory reorganization process in addition to the reinstatement process of stored memories. Kim et al. review recent human reconsolidation studies and discuss whether reconsolidation is a general property of all types of memory. They focus on the main controversial issues related to the general application of reconsolidation: whether declarative memory undergoes reconsolidation, and whether old memories can be reorganized after retrieval. Kim et al. suggest the possibility that the differing involvement of cortical circuits, and differing intensity levels of post-retrieval-onset interfering signals, may be the main factors behind the successful application of reconsolidation.

## Conclusion

Memory retrieval is important, in that it realizes stored information and influences our current behaviors. However, the articles in this Research Topic collectively suggest the view that retrieval is a dynamic process and that it involves more than simply replaying stored information. The articles support the contentions that the retrieval process dynamically interacts with other cognitive processes and that retrieval itself can act as a signal for strengthening or modifying memories. These studies of dynamic retrieval processes critically contribute to our comprehensive understanding of memory processes.

## Author Contributions

S-HL, XL, and MC wrote the manuscript. All authors contributed to the article and approved the submitted version.

## Funding

MC is funded by the National Science Foundation (Award 1947685) and SL is supported by the Basic Science Research Program through the NRF of Korea (2020R1A2C2007770).

## Conflict of Interest

The authors declare that the research was conducted in the absence of any commercial or financial relationships that could be construed as a potential conflict of interest.

## Publisher's Note

All claims expressed in this article are solely those of the authors and do not necessarily represent those of their affiliated organizations, or those of the publisher, the editors and the reviewers. Any product that may be evaluated in this article, or claim that may be made by its manufacturer, is not guaranteed or endorsed by the publisher.
